# Impact of lymphovascular invasion on otherwise low-risk papillary thyroid carcinomas: a retrospective and observational study

**DOI:** 10.1007/s12020-023-03475-8

**Published:** 2023-08-28

**Authors:** Francisca Marques Puga, Abir Al Ghuzlan, Dana M. Hartl, Mohamed-Amine Bani, Sophie Moog, Fabiana Pani, Ingrid Breuskin, Joanne Guerlain, Matthieu Faron, Desirée Denadreis, Eric Baudin, Julien Hadoux, Livia Lamartina

**Affiliations:** 1 Service d’oncologie Endocrinienne, Département d’imagerie, Gustave Roussy, Villejuif, France; 2 Serviço de Endocrinologia, Diabetes e Metabolismo, Centro Hospitalar Universitário de Santo António, Porto, Portugal; 3 Département de Biologie et Pathologie Médicale, Gustave Roussy, Villejuif, France; 4 Département d’anesthésie, Chirurgie et Radiologie Interventionelle, Gustave Roussy, Villejuif, France; 5 Service de Médecine Nucléaire, Département d’imagerie, Gustave Roussy, Villejuif, France

**Keywords:** Thyroid neoplasm, low-risk papillary thyroid carcinomas, lymphovascular invasion, venous vascular invasion, lymphatic vascular invasion, risk adjustment

## Abstract

**Purpose:**

Presence of venous vascular invasion is a criterion of intermediate risk of recurrence in papillary thyroid carcinoma (PTC). However, the presence and type of vascular invasion (lymphatic or venous) is often underreported and its impact on PTCs without other risk features remains unknown. The aim of this study was to evaluate the impact of both lymphatic and venous invasion on the risk of recurrence/persistence on otherwise low-risk PTCs.

**Methods:**

Retrospective study including patients with otherwise low-risk PTCs but with vascular invasion, diagnosed between 2013 and 2019. The persistence/recurrence during the follow-up was evaluated. Pathology was reviewed to confirm the presence of lymphovascular invasion and determine the type of invasion.

**Results:**

A total of 141 patients were included. Lymphovascular invasion was confirmed in 20.6%. After surgery, 48.9% (*N* = 69) of the patients received radioactive iodine (RAI). The median follow-up time was 4 [3–6] years. Overall, 6 (4.2%) patients experienced persistent/recurrent disease in the neck, including 3 with lymphovascular invasion, confirmed as “only lymphatic”. Overall, patients with tumors harboring lymphovascular invasion had sensibly more persistent/recurrence disease compared with those without lymphovascular invasion (10.3% vs 2.7%, *p* = 0.1), especially in the subgroup of patients not treated with RAI (20% vs 1.6%, *p* = 0.049) [OR 15.25, 95% CI 1.24-187.85, *p* = 0.033].

**Conclusion:**

Lymphovascular invasion, including lymphatic invasion only, is associated with a sensibly higher risk of persistent/recurrent disease in otherwise low-risk PTCs, namely in patients not treated with RAI. Lymphatic invasion could have a role in risk-stratification systems for decision making.

## Introduction

The incidence of thyroid cancer has been increasing over the last decades [1–7]. Almost the entire change has been attributed to an increase in the incidence of papillary thyroid carcinoma (PTC), responsible for about 85% of all diagnosed thyroid cancers [1, 3]. However, this increase has been reported as attributable to incidentally detected clinically occult cancers, due to the widespread use of imaging techniques and screening campaigns [2, 4, 5]. One of the contemporary challenges in thyroid cancer management is that of sparing lower-risk patients from overtreatment.

PTC spreads mainly through lymphatic vessels and some foci of lymphatic invasion by cancer cells can be observed; foci of venous invasion can be observed too when the tumors have a capsule [8, 9]. The question remains if the presence of one or more foci of lymphatic or venous invasion should prompt a different management in patients with tumors without other risk features.

According to the 2015 American Thyroid Association (ATA) guidelines, the presence of vascular invasion is a criterion for intermediate risk but it refers to venous invasion only [8]. While some studies demonstrated that vascular invasion is associated with worse clinical outcomes, others failed to prove it [9–16].

Furthermore, the criteria for diagnosing vascular invasion in thyroid carcinomas are poorly defined [8–11]. Vascular invasion has been used by pathologists to describe venous invasion exclusively, but also to describe lymphatic invasion, whose impact on PTCs remains also unknown [8–11]. Moreover, distinguishing venous invasion from lymphatic invasion is not always straightforward, making it sometimes impossible to provide a clear classification. Therefore, specific information on the type of vascular invasion presented is often absent from pathology reports.

Our aim was to evaluate the impact of both lymphatic and venous vascular invasion on the risk of disease persistence/recurrence of the patients with PTC and vascular invasion, otherwise fulfilling the criteria for low risk according to the 2015 ATA classification.

## Materials and methods

### Patients

A retrospective study of consecutive patients fulfilling the definition of low-risk PTC according to 2015 ATA guidelines except for the presence of vascular invasion was performed.

The pathology database of PTCs diagnosed at *Gustave Roussy*, *Villejuif*, *France* between 2013 and 2019, was analyzed. Patients with surgery and follow-up performed elsewhere were excluded. We selected consecutive patients with low-risk PTC features, according to 2015 ATA guidelines: patients with intrathyroidal tumors, clinically N0 and with no >5 micrometastases (<2mm) of cervical lymph nodes, no macroscopically incomplete tumor resection, no aggressive histology, and no distant metastases [8]. Patients with documented vascular invasion, with no other intermediate or high-risk features according to the ATA guidelines were also included [8]. Only the features available before RAI treatment were taken into account, as this information is the more relevant for decision making.

Subsequently, we examined the clinical records to confirm the information and collect relevant demographic and clinical data such as sex, age, type of surgery, pathological features, and occurrence of persistent or recurrent disease. The tumor stage was classified according to the American Joint Committee on Cancer TNM (AJCC/TNM) staging system, 8th edition. Patients with a follow-up of less than 3 years were excluded from the study.

All the patients participating in this study had total thyroidectomy or lobectomy by high-volume thyroid surgeons, after multidisciplinary discussion. All patients were submitted to a preoperative specialized neck ultrasonographic assessment of the neck [17, 18]. Prophylactic lymph node dissection was performed in some patients, according to multidisciplinary tumor board discussion and shared decision making with the patient or in case of patients included in the ESTIMABL3 trial and randomized in the prophylactic dissection arm of the study (NCT03570021). Radioactive iodine therapy (RAI) was used in selected patients after multidisciplinary discussion taking into account clinic-pathologic information available after surgery (serum markers assessment was not routinely performed to assist RAI decision) or in case of patients included in the ESTIMABL3 trial. Each RAI administration was coupled to stimulated thyroglobulin (Tg) and Tg antibodies (TgAb) measurement, and neck ultrasound and followed by iodine whole-body scan and a SPECT/CT.

### Follow-up and clinical outcomes

Follow-up visits were performed at 3 to 6 months from primary treatment, then yearly for the first 5 years and then every 2 years. During the follow-up period, laboratory tests were performed, including serum Tg and TgAb measurement. Neck ultrasound was also performed after 12 months of follow-up or in case of abnormal serum Tg or TgAb, and fine-needle biopsy was used in the presence of suspicious lymph nodes (with Tg measurement on washout fluid) or nodules in the neck area if clinically appropriate. For patients who received lobectomy only, follow-up was performed with the same time schedule by neck ultrasound.

The response to therapy was evaluated at 12–18 months after surgery and at the last follow-up visit and classified as “Excellent response” (normal imaging and Tg <0.2 ng/mL and negative TgAbs), “Indeterminate response” (indeterminate findings on imaging or Tg between ≥0.2 and <1 ng/mL or stable or declining TgAbs titers), “Biochemical incomplete response” (normal imaging and Tg ≥1 ng/mL or rising TgAbs titers), or “Structural incomplete response” (suspicious imaging regardless Tg and AbTg status), according to the 2015 ATA response to therapy classification [8]. The same Tg cutoffs were applied to the patients submitted exclusively to total thyroidectomy without RAI treatment. Patients submitted to lobectomy were considered as having an “Excellent response” if no suspicious lymph nodes or suspicious nodules were found on neck ultrasound evaluation. The occurrence of persistence/recurrence of disease at any time of the follow-up was also evaluated. The cumulative rate of persistent/recurrent disease was defined as any evidence of structural disease on imaging with or without abnormal biochemical findings after initial surgery.

Tg and TgAb were measured at the institutional laboratory using the Beckman DXi800 Access Thyroglobulin and Access Thyroglobulin Antibody II kits. Starting from July 1st 2020 the BRAHMS hTg sensitive KRYPTOR and the BRAHMS anti-Tgn KRYPTOR kits were used.

### Vascular invasion

A standardized pathology report is used for thyroid cancer diagnosis in *Gustave Roussy* since 2012 and includes the presence or absence of vascular invasion as required by the protocol of the College of American Pathologists [19].

An endocrine pathologist with >15 years of experience on thyroid cancer (AAG) reviewed all the slides of the patients with suspicious or documented vascular invasion on the original pathology report in order to confirm its presence and to class the type of invasion as lymphatic or venous. HES-stained sections were screened to confirm the presence of lymphovascular invasion and the type of vessels involved were described. We classified the foci of vascular invasion into four groups: “only lymphatic“, “only venous”, “mixed” or “impossible to classify”. Lymphatic vessel invasion included the presence of tumor tissue and/or psammoma bodies in lymphatic spaces, including lymphatic spaces within the tumor, but also at the periphery of the tumor, or somewhere else in the resection specimen, as described by Mete et al. and Wittekind [19, 20].

**Fig. 1:**
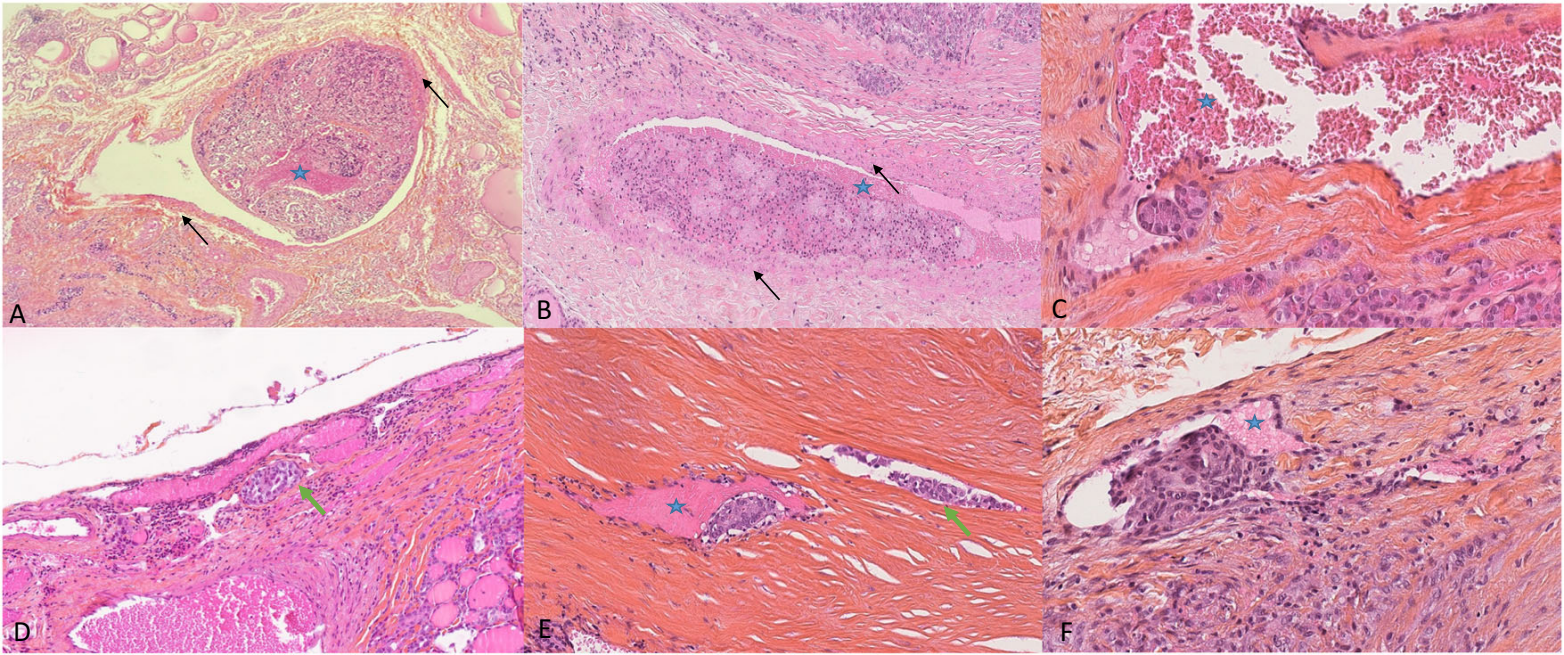
Different pathological types of vascular invasion in differentiated thyroid carcinomas. **A**, **B** Venous invasion: organized tumoral thrombus in a large venous space with presence of muscular fibers (black arrows **→**). **C** Endothelialized tumor deposits juxtaposed to the vessel wall. **D** Lymphatic invasion (green arrow) shows slit-like spaces filled partially with tumor cells. **E** Small tumor nodule within a thin vessel without red blood cells considered as a lymphatic invasion. **F** Small vessel with presence of tumor cells and partially filled with red blood cells (star) considered “impossible to classify”. The blue star shows the presence of red blood cells

For venous vascular invasion, the blood vessels are located outside the tumor, within the capsule, or outside the capsule. Invasion of a venous space is easily recognized by the presence of a visible layer of smooth muscle fibers in their wall. For small vessels, it is difficult to distinguish between capillary sized vascular spaces and lymphatics. Morphologically smaller vascular spaces usually have red blood cells within. Whenever it was impossible to do the distinction, the term “impossible to classify” was used. Some examples of these features are provided in Fig. 1.

The number of foci of each type of vascular invasion was also registered.

### Statistical analysis

Statistical analysis was conducted using SPSS software (Statistical Package for Social Sciences) version 27.0. For continuous quantitative variables, distribution normality was tested through histogram observation and kurtosis and skewness analysis. The results are presented as mean ± standard-deviation (SD) or median [interquartile range].

The goodness of fit *χ*^2^-test was used to compare frequencies between the categorical variables. The student’s t-test for independent variables and the Mann-Whitney test were used to compare continuous variables with normal and non-normal distribution between groups, respectively. A logistic regression model was performed to evaluate vascular invasion as a predictor of persistent/recurrent disease, adjusting for potential confounders using a stepwise regression with a forward inclusion approach. Results will be presented as hazard ratios with 95% confidence intervals. A two-sided *p* value < 0.05 was considered statistically significant.

This study was approved by the local Ethics committee of *Gustave Roussy*. Patient consent was waived by the Ethics Committee due to the retrospective nature of the study and full data anonymization.

## Results

### Sample Characteristics

**Fig. 2:**
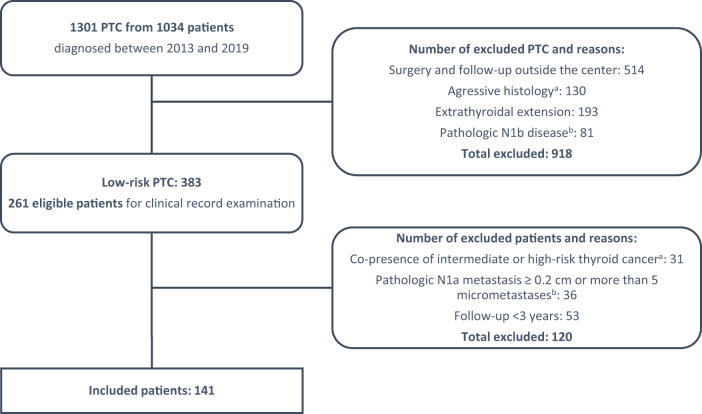
Flowchart of the study illustrating the eligible and included patients. PTC: Papillary thyroid carcinoma.^a^ According to the ATA risk stratification system. ^b^According to the American Joint Committee on Cancer TNM (AJCC/TNM) stating system, 7th edition

During the study period, a total of 1301 PTCs were diagnosed at *Gustave Roussy*. We examined the clinical records of 261 eligible patients. Finally, 141 patients were included in the study cohort (patient selection process is depicted in Fig. 2). The mean age at initial diagnosis was 47.9 ± 14.4 years and 75.9% of the patients were female (*N* = 107). The majority had total thyroidectomy (87.9%, *N* = 124) and central neck lymph node dissection was performed in 58.9% (*N* = 83) of the patients. The clinicopathologic characteristics of the patients are presented in Table 1.

**Table 1:** Clinicopathologic characteristics of the patients

Age (years)	47.9 ± 14.4
Female gender (%, *n*)	75.9% (107/141)
Surgery (%, *n*)	
Total thyroidectomy	87.9% (124/141)
Lobectomy	4.3% (6/141)
Lobectomy + isthmusectomy	7.8% (11/141)
Central lymph node dissection (%, *n*)	58.9% (83/141)
Multifocality (%, *n*)	24.1% (34/141)
Largest size of the dominant tumor (mm)*	13 [10–21]
Histology (%, *n*)	
Classic PTC	51.8% (73/141)
Follicular variant	34.0% (48/141)
Others^a^	14.2% (20/141)
Capsular invasion (%, *n*)	41.1% (58/141)
Lymphovascular invasion confirmed by slide revision (%, *n*)	20.6% (29/141)
Number of invasion foci*	2 [1–3]
Type of invasion (%, *n*)	
* Only lymphatic*	51.7% (15/29)
* Only venous*	20.7% (6/29)
* Mixed*	13.8% (4/29)
* Impossible to classify*	13.8% (4/29)
TNM classification^b^ (%, *n*)	
T	
T1a	40.4% (57/141)
T1b	33.3% (47/141)
T2	22.0% (31/141)
T3a	4.3% (6/141)
N	
N0	52.5% (74/141)
Nx	41.8% (59/141)
N1a	5.7% (8/141)
M	
M0	48.9% (69/141)
Mx	51.1% (72/141)
RAI after surgery (%, *n*)	48.9% (69/141)
Dose	
* 30 mCi*	85.5% (59/69)
* 100 mCi*	14.5% (10/69)
Preparation	
* Thyroid hormone withdrawal*	10.1% (7/69)
* Recombinant human TSH*	89.9% (62/69)

Data are presented as mean ± standard deviation, unless otherwise indicated by * corresponding to data presented as median, 25th and 75th percentiles

*PTC* papillary thyroid carcinoma, *RAI* radioactive iodine therapy, *TSH* thyroid stimulating hormone

^a^Warthin-like variant, oncocytic variant or with no other specification

^b^American Joint Committee on Cancer TNM (AJCC/TNM) stating system, 7th edition

On histology, 24.1% (*N* = 34) had multifocal low-risk PTC. The median size of the largest tumor was 13 [10−21]mm, with 23.4% (*N* = 33) of the patients having a microcarcinoma (i.e. ≤10 mm). Invasion of tumor capsule was present in 77.3% (*N* = 58) of the 75 patients with an encapsulated tumor. On pathology reports, lymphovascular invasion was listed as present in 36 (25.5%) and suspicious in 10 (7.1%) patients. Eight patients (5.7%) had lymph node metastases at the time of the diagnosis, all of them with N1a disease and less than 5 metastatic lymph nodes of <2 mm (micrometastases). After surgery, 48.9% (*N* = 69) of the patients were treated with RAI. The most frequently used RAI activity was 30 mCi (85.5%, *N* = 59) after recombinant human TSH injection. Patients having RAI after surgery included all the patients with lymph node metastases and had a significantly larger tumor size compared with the patients not treated with RAI [20 (25–27) mm vs 10 (8–14) mm, *p*<0.001]. Additionally, patients receiving RAI had a significantly higher prevalence of lymphovascular invasion [27.5% (19/69) vs 13.9% (10/72), *p* = 0.047]. No other differences were found between the two groups (Supplementary Table 1). Post therapeutic iodine whole-body scan and SPECT/CT revealed RAI-avid disease in the neck outside the thyroid bed consistent with RAI avid lymph node metastases in 2 patients (1.4%), one of them with visible suspicious lymph nodes on neck ultrasound.

### Clinical outcomes of the cohort

The median follow-up time was 4 [3−6] years, ranging from 3 to 8 years. Two patients died from unrelated causes during the follow-up time. Twelve to eighteen months after surgery, the majority of the patients had an “Excellent response” (66%, *N* = 93) or an “indeterminate response” (31.9%, *N* = 45), while 2 (1.4%) patients had a “Structural incomplete response” and one (0.7%) a “Biochemical incomplete response”. At the last follow-up visit, none of the patients had “Structural incomplete response”. Four (2.8%) patients had a “Biochemical incomplete response” at the last follow-up visit, one due to abnormal Tg value (Tg = 2.8 ng/mL) and 3 due to a rise in TgAb titers (undetectable Tg with TgAb of 46, 57 and 1676 UI/mL). A recent TgAb assay modification at our center might explain this modification. The clinical outcomes of the follow-up according to RAI treatment are presented in supplementary data (Supplementary Table 2).

Overall, 6 (4.2%) patients experienced persistent/recurrent disease at any time after initial surgery, all in neck lymph nodes. Their characteristics are summarized in Table 2. All of them had an “Excellent response” at the time of the last follow-up visit.

**Table 2:** Clinical characteristics of the patients that experienced persistent/recurrent disease during the follow-up

Sex	PTC stage^a^	Initial treatment	Persistence/Recurrence	Diagnosis	Additional treatments	Last-follow-up visit
Female	T1bN0	Surgery + RAI	Persistent disease	Neck RAI avid disease after initial RAI	No	Excellent response (5 years)
Female^b^	T2Nx	Surgery + RAI	Persistent disease	Neck RAI avid disease after initial RAI	RAI	Excellent response (3 years)
Male	T1bN0	Surgery + RAI	Persistent disease	Incidental diagnosis of persistent locoregional disease 4 months after surgery, when submitted to additional neck surgery due to laryngeal carcinoma	No	Excellent response (3 years)
Male	T1bN0	Surgery	Persistent disease	Incidental diagnosis of persistent locoregional disease 5 months after surgery, following cervical abscess investigation	Surgery + RAI	Excellent response (6 years – died due to unrelated causes)
Female^b^	T1bN0	Surgery	Recurrent disease	Cytology diagnosis of locoregional disease after Tg elevation and suspicious neck ultrasound 15 months after surgery	RAI	Excellent response (4 years)
Female^b^	T1aN0	Surgery	Recurrent disease	Cytology diagnosis of locoregional disease after suspicious neck ultrasound, despite undetectable level of Tg, 6 years after surgery	Surgery	Excellent response (8 years)

*PTC* papillary thyroid carcinoma, *RAI* radioactive iodine therapy, *Tg* thyroglobulin

^a^American Joint Committee on Cancer TNM (AJCC/TNM) stating system, 7th edition

^b^Lymphatic invasion present

### Lymphovascular invasion and risk of recurrence/persistence

Lymphovascular invasion was described as present or suspicious in 25.5% (*N* = 36) and 7.1% (*N* = 10) of the original reports, respectively. After the slides were reviewed, lymphovascular invasion was confirmed in only 20.6% (*N* = 29). The remaining cases, corresponding to 30% (11/36) of the cases with lymphovascular invasion on the original reports and 60% of the cases with suspicious lymphovascular invasion (6/10) were finally classified as without lymphovascular invasion after slide revision.

The median number of invasion foci was 2 [1−3] foci, with one patient with innumerable foci. Among the patients with lymphovascular invasion, the kind of invasion was classified as “only lymphatic” in 15 (51.7%) patients, “only venous” in 6 (20.7%), “mixed” in 4 (13.8%) and “impossible to classify” in 4 (13.8%) patients (Table 1). Three of the 6 patients that experienced persistence/recurrence during the follow-up time had lymphovascular invasion, all confirmed as “only lymphatic” by pathologic revision. Two of them had only one invasion foci and the other one innumerable foci.

**Fig. 3:**
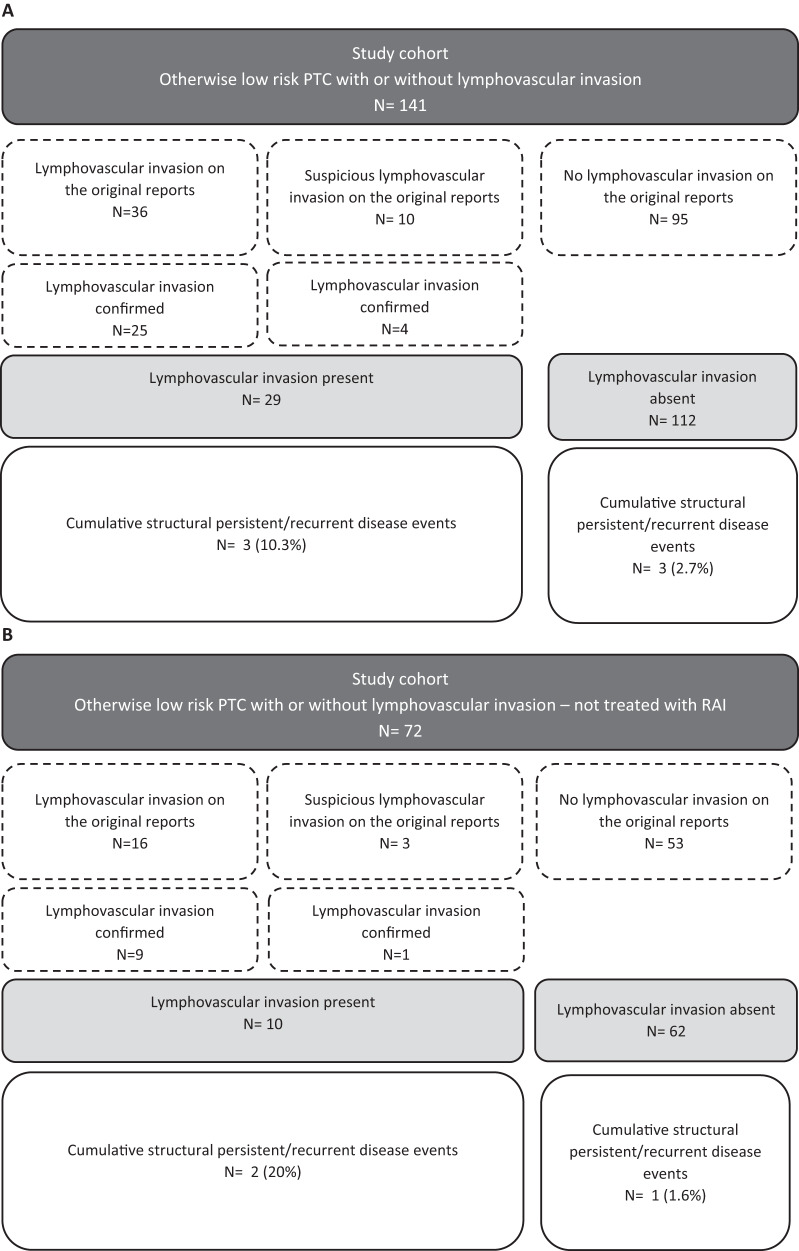
Comparison between patients with and without lymphovascular invasion. **A** Whole cohort. **B** Patients not treated with radioiodine

The comparison between patients with and without lymphovascular invasion showed that persistent/recurrent disease was sensibly more frequent in patients with confirmed lymphovascular invasion [10.3% (3/29) vs 2.7% (3/112), *p* = 0.1] [OR 4.19, 95% CI 0.80–21.97, *p* = 0.090] (Fig. 3, panel A). Similarly, the comparison between patients with and without recurrent disease showed that lymphovascular invasion tended to be more frequent in the recurrence group (50.0% vs 19.3%, *p* = 0.070). There were no differences regarding age, gender, multifocality, size of the tumor, nodal involvement, RAI use after surgery or follow-up length (Table 3).

**Table 3:** Comparison of the clinical characteristics between patients with and without persistent/recurrent disease during the follow-up time

	Cumulative persistent /recurrent disease *N* = 6	No evidence of disease *N* = 135	*p*
Age (years)	54.7 ± 20.9	47.6 ± 14.1	0.241
Female gender (%, *n*)	66.7% (4/6)	76.3% (103/135)	0.592
Lymph node dissection (%, *n*)	83.3% (5/6)	57.8% (78/135)	0.216
Multifocality (%, *n*)	16.7% (1/6)	24.4% (33/135)	0.667
Largest size of the dominant tumor (mm)*	12 [11−12]	14 [10–22]	0.468
Lymphovascular invasion (%, *n*)	50.0% (3/6)	19.3% (26/135)	0.070
Nodal involvement (%, *n*)	0.0% (0/6)	5.9% (8/135)	0.542
RAI after surgery (%, *n*)	50.0% (3/6)	48.9% (66/135)	0.958
Follow-up time (years)*	4 [3–6]	4 [3–6]	0.678

Data are presented as mean ± standard deviation, unless otherwise indicated by * corresponding to data presented as median, 25th and 75th percentiles

*RAI* radioactive iodine therapy

Interestingly, when analyzing the 72 patients not treated with RAI separately, patients with lymphovascular invasion had a higher rate of persistent recurrent disease compared with patients without lymphovascular invasion [20% (2/10) vs 1.6% (1/62), *p* = 0.049] [OR 15.25, 95% CI 1.24–187.85, *p* = 0.033] (Fig. 3, B).

Due to the scarce number of events, a multivariate analysis was not performed.

## Discussion

Our study shows that lymphovascular invasion is associated with a modestly higher risk of persistent/recurrent disease (cumulative rate of persistent/recurrent disease of about 10%) in PTC patients without other risk features, consistent with intermediate risk of recurrence, compared with those patients with low-risk PTC without lymphovascular invasion (cumulative rate of persistent/recurrent diseases <3%). As for the kind of invasion, all the persistent/recurrent disease events occurred in cases with lymphatic invasion only. As expected, all persistent/recurrent disease events were observed in neck lymph nodes. Among patients not treated with RAI, lymphovascular invasion was associated with a higher rate of persistent/recurrent disease.

Papillary thyroid carcinoma is a common disease among adults [1, 3]. The broad spectrum of aggressiveness encourages the adoption of a risk-based approach, in order to minimize overtreatment and provide aggressive therapy when warranted [6–8]. Vascular invasion is a criterion of intermediate risk and refers to venous invasion only according to the 2015 ATA guidelines [8]. Nonetheless, this pathological feature is often underreported and the term vascular invasion can be used to describe venous invasion exclusively, but also to describe lymphatic invasion, whereas sometimes it is impossible to classify unequivocally between these two conditions even by very experienced pathologists [8–11]. Herein we explored the potential impact of lymphovascular invasion on “otherwise low-risk” PTCs suggesting a role of lymphatic invasion alone for PTC prognostication.

This study has some limitations. The population sample is relatively small and the low number of persistent/recurrent disease events hampered a multivariate analysis. Nonetheless, the studied population was very uniform with regard to the follow up schedule and well characterized thanks to the pathologic evaluation performed in a referral center. The low number of events observed is in accordance with the one expected in a lower-risk population. Another weakness of the study is the limited follow-up time of 4 years, as late recurrences are described in low-risk PTCs [21]. However, at least half of the recurrences are detected within the first 3 years of follow-up and >75% within the first 5 years [22]. Furthermore, slides from patients without lymphovascular invasion on the original reports were not reviewed by the pathologist, making it impossible to exclude misinterpretation of these cases. It is worth note that the original reports were all performed in the same institution using a standardized pathologic report including the vascular invasion item, limiting the risk of underreporting. Most of the cases that were not confirmed were indicated as suspect vascular invasion on the original reports indicating rather an attitude to over reporting this feature.

Moreover, some patients were subjected to more scrutiny than others, such as patients treated with RAI explored with post therapeutic whole body scan and SPECT/CT, justifying some of the persistent/recurrent events. Nevertheless, lymphovascular invasion was shown to be a risk factor for persistent/recurrent disease, particularly in RAI not treated and not in RAI treated patients.

In our sample, patients not treated with RAI had smaller primary tumors and no lymph node metastases. Nevertheless, our results show a higher recurrence rate when lymphovascular invasion was present in this group of patients, highlighting the role of lymphovascular invasion, as a prognostic factor in patients with no other risk features. We acknowledge that the number of events was too small to drive firm conclusions and that these results need further confirmation on larger independent cohorts.

Interestingly, all patients experiencing structural persistent/recurrent PTC in our population achieved excellent response at last follow-up assessment, suggesting no need for upfront aggressive treatment as the rare cases experiencing persistent/recurrent disease can undergo salvage surgery or RAI treatment later. Therefore, the role of lymphovascular invasion in decision making might be that of orienting the choice of the frequency and the tools used for follow-up assessments. Indeed, lymphatic invasion can be associated with a risk of lymph node metastasis, whereas venous invasion can be associated with hematogenous dissemination and risk of distant metastasis [11].

The number of foci of invasion is a well-established prognostic feature of follicular and oxyphylic thyroid cancer [23]. In our study it was not possible to determine if the number of foci was relevant for PTC prognostication, due to the low number of events. Among the patients with recurrence and lymphatic invasion one patient had very extensive invasion, with innumerable foci, while the other two had only one focus of invasion.

Lymphovascular invasion is frequently not fully described and often absent from pathology reports, in part due to the poorly defined criteria for diagnosis [8–11]. In our sample, the review of the slides by an experienced pathologist led to discordance in some patients, with about 5% of the cases not confirmed as harboring lymphovascular invasion. Unfortunately, it was not possible to perform a double side review by a second experienced pathologist in order to provide data on interobserver variability and we acknowledge that these results might be not reproductible in a real life setting. Nevertheless, our results are in accordance with a previous study that observed a major discordance of 21% between initial and second-opinion histopathologic diagnosis in patients diagnosed with differentiated thyroid carcinoma [24]. The smaller discordance observed herein could be explained by the fact that all original pathologic diagnoses were made by our institutional pathologists. Our study highlights the importance of reporting both lymphatic and venous vascular invasion in pathology reports and also emphasizes the role of experienced pathologists. Therefore, well defined criteria for diagnosis of lymphovascular invasion are needed, and reports should mention lymphatic and venous invasion separately when possible. We acknowledge that the very high level of expertise of the pathologist may render our results hard to reproduce in the real-life setting. Nonetheless, controversial pathologic features with scarce reproducibility such as minimal extra thyroidal extension are part of ATA risk stratification classification.

In conclusion, our study suggests that lymphovascular invasion, including lymphatic invasion only, is associated with a modestly higher risk of persistent/recurrent disease in otherwise low-risk PTC, namely in patients not treated with RAI. Therefore, lymphatic invasion might be included in risk-stratification systems to refine decision making in this population and might guide the choice of follow-up tools to be employed for these patients.

Further research is needed to confirm our results.

### Supplementary information

Supplementary data
