# Supplementary material for: Impact of lymphovascular invasion on otherwise low-risk papillary thyroid carcinomas: a retrospective and observational study
Source: Endocrine. 2023 Aug 28;83(1):150–9. doi: 10.1007/s12020-023-03475-8 (PMC10805903; doi:10.1007/s12020-023-03475-8)
Supplement: Supplementary file 1 — Supplementary data [file 12020_2023_3475_MOESM1_ESM.docx]

**Supplementary data**

**Table 1.** Comparison of the clinical characteristics between patients treated and not treated with RAI after surgery (supplementary material)

|  | **RAI** | **No RAI** | **p** |
| --- | --- | --- | --- |
| Age (years) | 48.7 ± 14.0 | 47.2 ± 14.8 | 0.557 |
| Female gender (%, n) | 75.4% (52/69) | 76.4% (55/72) | 0.890 |
| Lymph node dissection (%, n) | 66.7% (46/69) | 51.4% (37/72) | 0.067 |
| Multifocality (%, n) | 29.0% (20/69) | 19.4% (14/72) | 0.184 |
| Largest size of the dominant tumor (mm)* | 20 [15 - 27] | 10 [8 - 14] | <0.001 |
| Lymphovascular invasion (%, n) | 27.5% (19/69) | 13.9% (10/72) | 0.047 |
| Nodal involvement (%, n) | 11.6% (8/69) | 0.0% (0/72) | 0.003 |

Data are presented as mean ± standard deviation, unless otherwise indicated by * corresponding to data presented as median, 25th and 75th percentiles. RAI: radioactive iodine therapy.

**Table 2.** Follow-up clinical outcomes of the patients (supplementary material)

|  |  |  | | |
| --- | --- | --- | --- | --- |
| Response to therapy (%, n)^a^  12-18 months after surgery  *Excellent response*  *Indeterminate response*  *Biochemical incomplete response*  *Structural incomplete response*  At the last follow-up visit  *Excellent response*  *Indeterminate response*  *Biochemical incomplete response*  *Structural incomplete response* | | No RAI  47.2% (34/72)  50.0% (36/72)  1.4% (1/72)  1.4% (1/72)  47.2% (34/72)  51.4% (37/72)  1.4% (1/72)  0.0% (0/72) | | RAI  85.5% (59/69)  13.0% (9/69)  0.0% (0/69)  1.4% (1/69)  82.6% (57/69)  13.0% (9/69)  4.3% (3/69)  0.0% (0/69) |
| Cumulative persistent/recurrent disease at any time of the follow-up (%, n) | | | 4.2% (6/141) | |

^a^ According to ATA PTC response to therapy classification
